# Precision in Complexity: Efficacy of VSP and Mock Surgery in Stock Prosthetic Reconstruction for TMJ Re-Ankylosis

**DOI:** 10.3390/cmtr19040041

**Published:** 2026-10-12

**Authors:** S. Hemanth Kumar, Shadab Mohammad, Vibha Singh, Geeta Singh

**Affiliations:** Department of Oral and Maxillofacial Surgery, King George’s Medical University, Lucknow 226003, India; shadab31aug@yahoo.com (S.M.); vibhasinghraghuvanshi@gmail.com (V.S.); drgeetasinghkgmc@gmail.com (G.S.)

**Keywords:** TMJ ankylosis, VSP, MOCK surgery, stock prosthesis, alloplastic total joint replacement

## Abstract

Introduction: Temporomandibular joint ankylosis (TMJA) management has evolved from gap arthroplasty to total joint replacement (TJR). TMJ re-ankylosis presents a significant challenge in achieving long-term treatment success. This study evaluates the effectiveness of virtual surgical planning (VSP) guided stock TJR in TMJ re-ankylosis. Materials and methods: Prospective, single-arm clinical interventional study with 10 patients (mean age 20.3 ± 1.37 years, range 18–28 years; 5 males, 5 females) with TMJ re-ankylosis were prospectively evaluated. Preoperative workflow integrated VSP for 3D mass assessment, cutting guide fabrication, and ramal osteoplasty mapping, followed by physical 1:1 stereolithographic mock surgery validation prior to alloplastic stock TJR (OrthoMax^®^). Functional outcomes were analyzed over a mean follow-up of 60 months. Result: This study showed statistically significant improvement in maximum interincisal opening (MIO) from 1.83 mm to 37.83 mm (*p* < 0.001), bite force from 0 kg to 10.31 kg (*p* < 0.001), masticatory efficiency, and diet consistency post-stock-TJR with a mean follow-up of 60 months. Mandibular range of movements and quality of life improved markedly with minimal postoperative complications. Conclusions: This study highlights the clinical success of the dual validation protocol by integrating VSP-guided stock-TJR in TMJ re-ankylosis. Mock surgery verified digital precision with intraoperative flexibility, ensuring optimal prosthesis fit, restored mandibular function, improvement in quality of life (QoL) and functional rehabilitation. With ongoing advancements, the VSP-guided stock-TJR protocol can be considered effective in TMJ re-ankylosis.

## 1. Introduction

TMJA is a pathological condition leading to jaw hypomobility causing functional, aesthetic, and psychological issues. Long-term treatment success in TMJ re-ankylosis is challenging, with management evolving from conventional gap arthroplasty to TJR. In re-ankylosis, autogenous grafts such as costochondral grafts (CCG) frequently suffer from unpredictable resorption, donor-site morbidity, or heterotopic bony re-ankylosis due to scarred recipient beds and compromised vascularity [1], supporting the rationale for alloplastic TJR. Today, alloplastic TJR is increasingly recognized as the gold standard for managing end-stage TMJ disorders [2].

Modern TJR systems are designed to restore function, alleviate pain, and improve patients’ QoL. As of today, TMJ Concepts, Biomet Microfixation and Nexus CMF are FDA-approved prosthetic systems for TJR [3]. A Stock TMJ Replacement System provides a stock prosthesis that resembles a ‘‘ball and socket’’-type prosthetic joint resembling a hip implant. This study evaluates a dual validation protocol incorporating VSP along with mock surgery-guided stock-TJR for TMJ re-ankylosis. The primary objective of this study is to assess the efficacy of the dual validation protocol in the management of TMJ re-ankylosis.

## 2. Materials and Method

**Figure 1:**
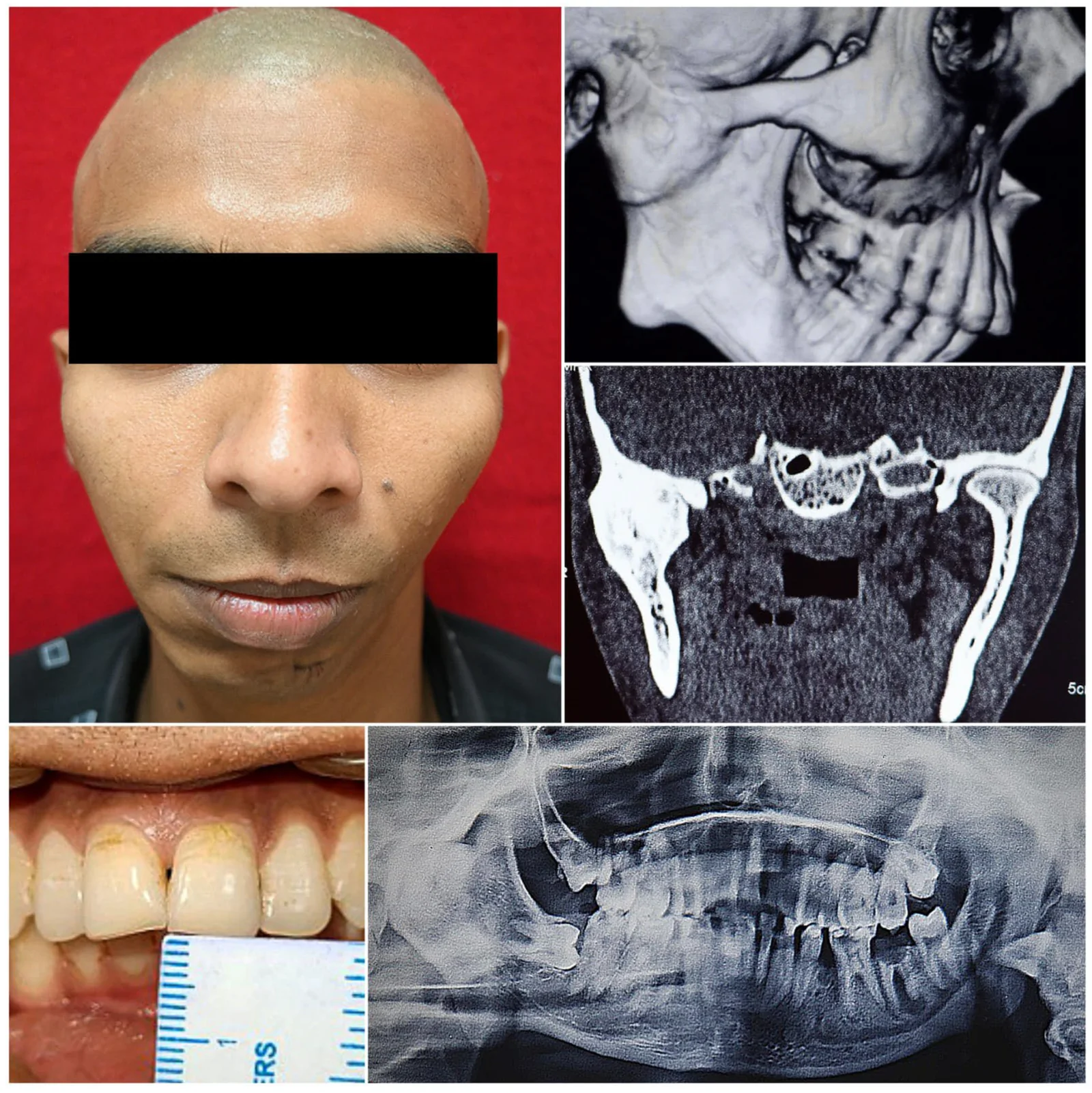
Preoperative clinical and radiographic image showing right TMJ re-ankylosis: (**Top Left**) Frontal clinical photograph demonstrating facial asymmetry and mandibular deviation to the affected side; (**Top Right**) 3D-CT illustrating the dense bony fusion of the right TMJ; (**Middle Right**) Coronal CT section showing the extensive ankylotic block and its close anatomical proximity to the skull base; (**Bottom Left**) Clinical photograph demonstrating preoperative MIO = 1 mm; (**Bottom Right**) Preoperative OPG showing complete obliteration of the right TMJ space with surrounding sclerotic bone.

This prospective, single-arm clinical interventional study was conducted in the Department of Oral and Maxillofacial Surgery at King George’s Medical University, Lucknow, India. Ten patients with TMJ re-ankylosis necessitating alloplastic joint reconstruction meeting the inclusion criteria were prospectively enrolled, with a mean post-reconstruction follow-up of 60 months. During formal preoperative counseling, alternative surgical options—including autogenous costochondral grafting and custom patient-specific implants (custom TJR)—were explained in detail. Stock alloplastic TJR using the dual-validation workflow was chosen due to the prohibitive financial costs and prolonged manufacturing lead times of custom PSI systems. Written informed consent was obtained from all participants. Baseline demographic and diagnostic parameters are detailed in Table 1 [Figure 1].

Inclusion Criteria

- TMJ re-ankylosis.
- Medically fit patient.

Exclusion Criteria

- TMJA.
- Medically compromised patients.

**Figure 2:**
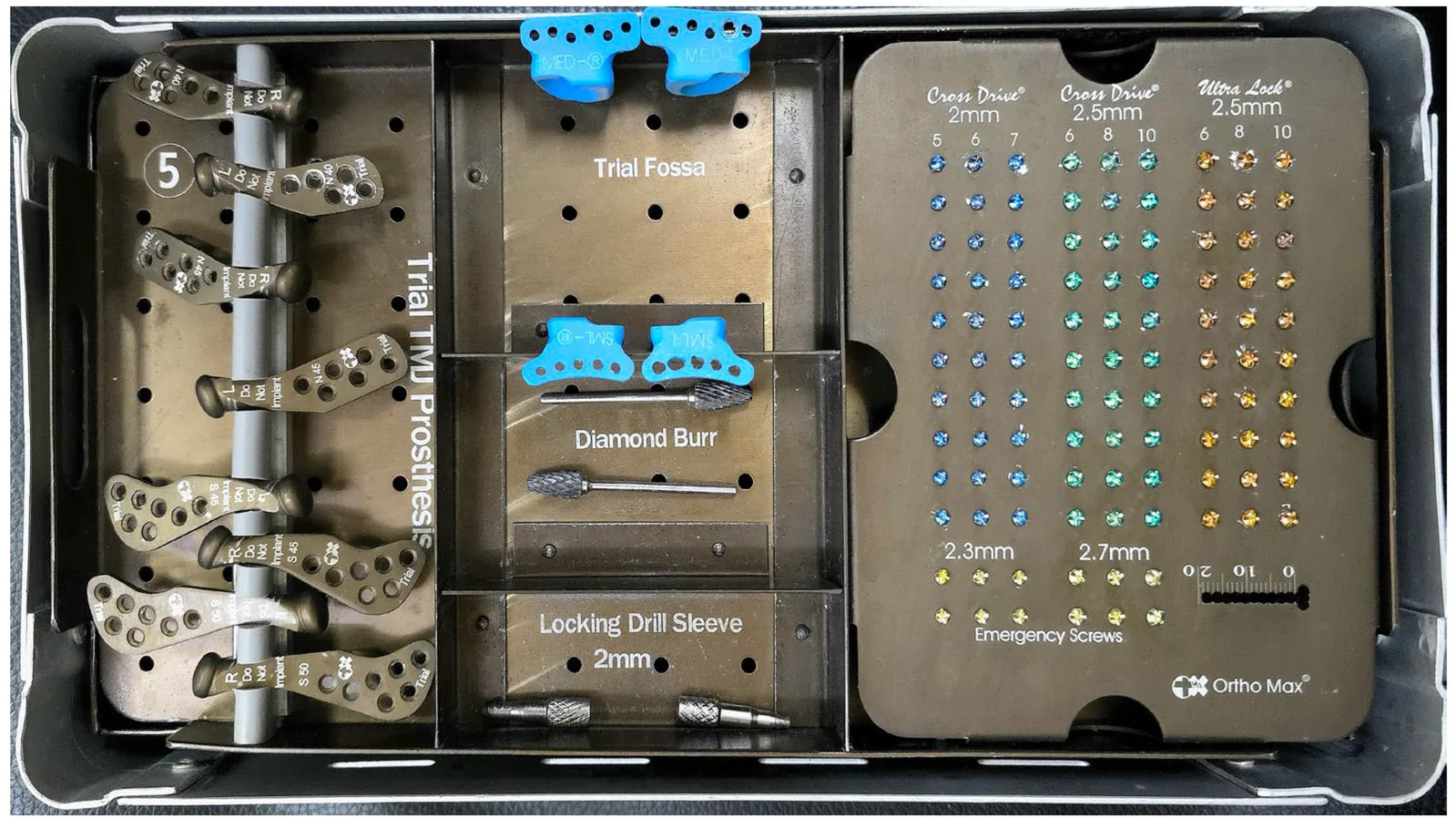
Stock TMJ Replacement System.

TMJ Stock Prosthesis [Figure 2]

OrthoMax^®^ Stock TMJ Replacement System (OrthoMax Manufacturing Co., Pvt. Ltd., Vadodara, Gujarat, India), manufactured under ISO 13485:2016 [4] standards and approved by the Central Drugs Standard Control Organisation (CDSCO/Indian FDA), was used. Components include:

- Condyle component made of medical-grade V titanium alloy (Ti6Al4V) and available in standard and narrow 40 mm, 45 mm, and 50 mm sizes. The bone-contacting surface is milled for better osseointegration.
- Fossa component fabricated from medical-grade Ultra High Molecular Weight Polyethylene (UHMWPE), available in small and medium sizes.
- To mitigate creep, cold flow, and screw-head pull-through within the polymeric fossa under masticatory loads, a form-fitted Grade II commercially pure titanium fossa washer was secured across the superior fixation flange.
- Screws made of titanium grade V attach the condyle and fossa components to the bone.

**Figure 3:**
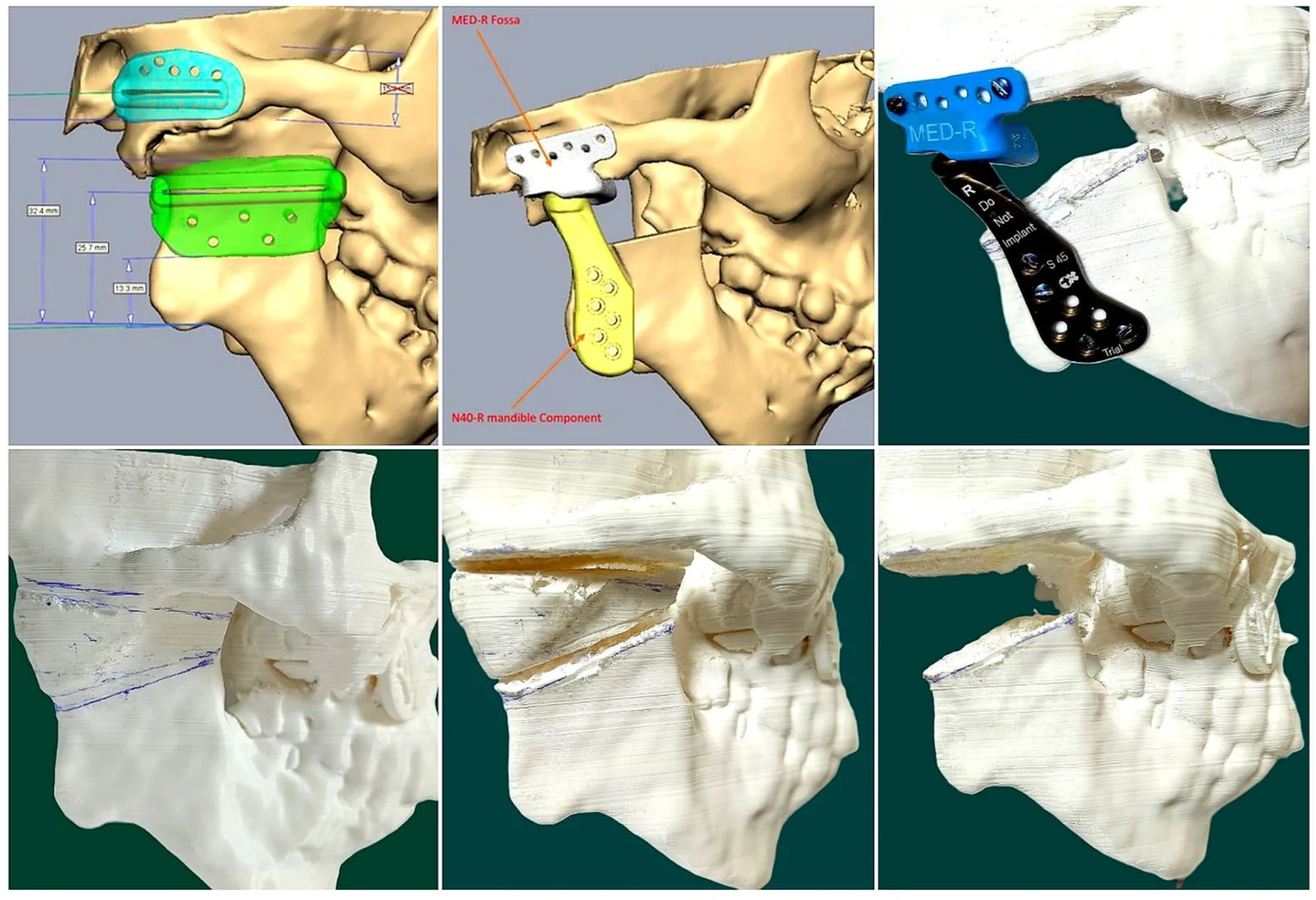
Dual-validation protocol integrating VSP and physical mock surgery: (**Top Left**) Digital STL segmentation and osteotomy planning; (**Top Center**) Virtual selection, and positioning of the stock glenoid fossa and condylar component; (**Top Right** & **Bottom Row**) Physical validation performed on a 1:1 3D-printed stereolithographic model showing cutting guide placement, bone resection rehearsal, and confirmation of trial fitting, prosthesis adaptation prior to live surgery.

Dual-Validation Protocol (VSP and Physical Mock Surgery) [Figure 3].

High-resolution CT scans (0.625 mm slice thickness) were reconstructed into 3D stereolithography (STL) files using Mimics Innovation Suite (Version 24.0, Materialise, NV, Leuven, Belgium). Virtual osteotomy planes were demarcated to ensure an 8 mm supra-osteotomy clearance from the skull base and a 15 mm condylar gap. Patient-specific ramal cutting guides were designed and 3D printed. 3D topographical analysis of the lateral ramus was conducted by digitally superimposing the stock condylar plate geometry onto the deformed ramal cortex; areas demonstrating bone-plate contact clearance <80% or cortical steps >1.0 mm were mapped for ostectomy. The digital plan was subsequently validated physically on a high-resolution 1:1 stereolithographic model. Osteotomies, cutting guide seatings, lateral cortical osteoplasties, and trial prosthesis insertions were performed sequentially on the model. The planned vs. mock-resected gap dimensions achieved a non-significant discrepancy (15.0 mm vs. 15.1 ± 0.3 mm, *p* > 0.05), with a 100% component size concordance rate between mock surgery and final intraoperative implantation.

Surgical procedure

**Figure 4:**
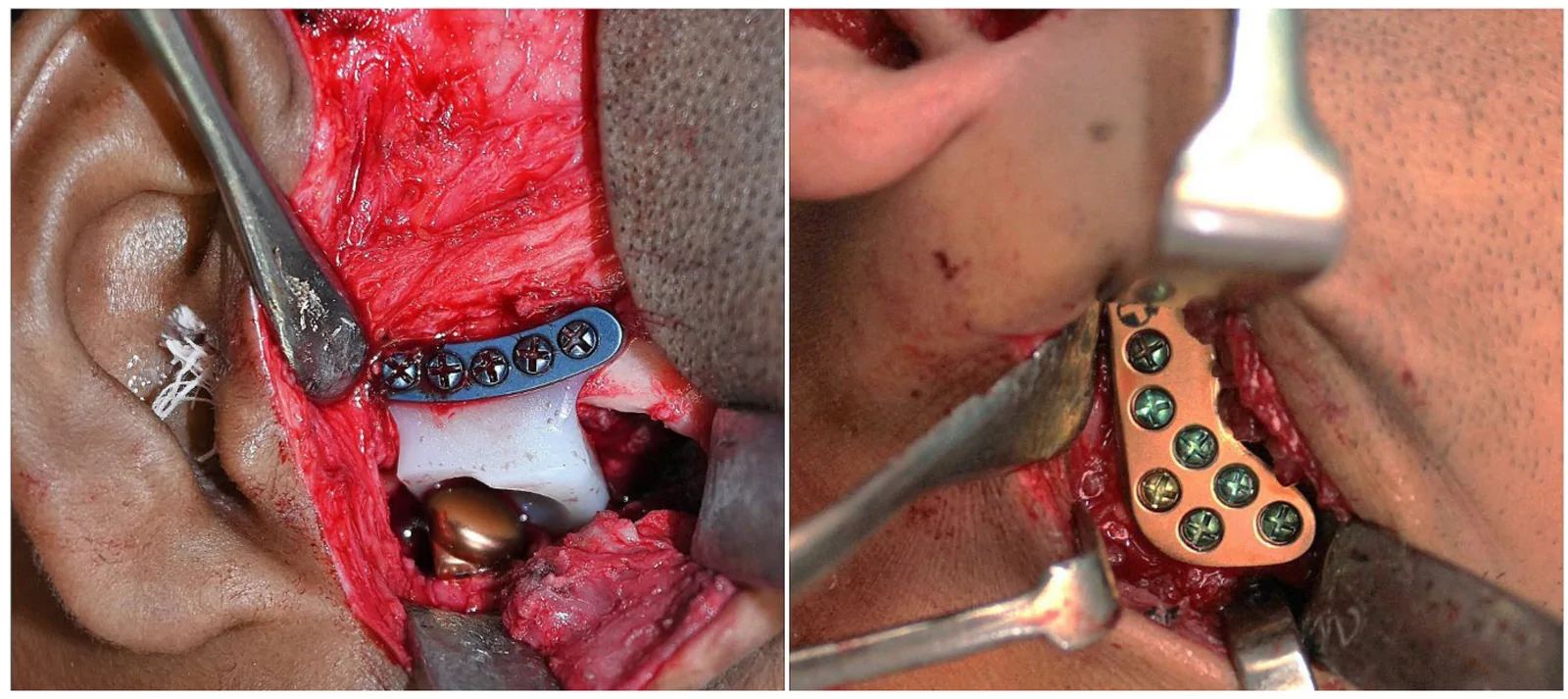
Intraoperative image showing glenoid fossa and ramal component in situ.

- All patients were operated on under hypotensive general anesthesia using aseptic standard operating protocol. Guided resections and ramal surface recontouring were executed in direct concordance with the pre-validated mock surgical plan.
- Al-Kayat Bramley approach for osteoarthrectomy and fossa component fixation. Cutting guide fixed and 15 mm condylar gap created. Mandibular movements assessed and MIO recorded. Submandibular approach for ramal surface modifications and condyle component fixation.
- Preoperative angiographic embolization was not required in any patient. Strict subperiosteal dissection to safeguard the maxillary artery and pterygoid venous plexus. Local hemostatic measures were used.
- Fossa component was positioned parallel to FH plane and stabilized with 6-screws. Intermaxillary fixation done.
- Ramus surface altered to achieve best adaptation of condyle component; stabilized parallel to posterior border of ramus with at least 10 screws, as posteriorly as possible in the fossa to achieve some degree of ‘pseudo–translation’ [Figure 4].
- Prosthesis function verified by assessing range of motion and MIO. Standard precautions to reduce periprosthetic joint infection (PJI) were taken.
- All the patients followed gradual postoperative mouth-opening exercise protocol.

Outcome Assessment and Reliability: Clinical outcomes were evaluated at scheduled intervals (Preop, Immediate Postop, 1st week, 2nd week, 4th week, 3rd month, 6th month, 1st year, 3rd year, 5th year). MIO (mm), lateral excursion (mm), and protrusion (mm) were measured using a calibrated scale. Bite force was recorded using a calibrated digital gnathodynamometer (accuracy ± 0.1 kg); each test was repeated three times at 1 min intervals, and the mean value was documented. Facial nerve function was graded using the House–Brackmann (HB) scale. Patient-reported quality of life was recorded using an adapted 10-domain Oral Health Impact Profile/TMJ-QoL questionnaire. The tool systematically captures 10 core domains: mouth opening, chewing ability/dietary intake, facial symmetry, occlusal stability, chin position, physical well-being, social well-being, psychological well-being, aesthetic satisfaction, and overall functional rehabilitation.

Responses were recorded on a 3-point Likert scale (Deteriorated, No Change, Improved, etc.) preoperatively and at sequential follow-up intervals up to final follow-up. All clinical measurements were recorded by a single clinical examiner.

Statistical Analysis: Statistical analysis was conducted using IBM SPSS Statistics (Version 23.0, IBM Corp., Armonk, NY, USA). Continuous longitudinal variables (MIO, bite force) were expressed as mean ± standard deviation (SD) and 95% confidence intervals (95% CI); sequential differences were evaluated using paired Student’s *t*-tests. A post hoc power calculation (α = 0.05, effect size d = 8.78) confirmed >99% statistical power with *n* = 10 subjects. Categorical and ordinal data (sex, Sawhney type, HB facial nerve grades, diet consistency, jaw deviation, and QoL parameters) were summarized as numbers and percentages (%) and analyzed using the Chi-Square (χ^2^) test. A two-tailed *p*-value < 0.05 was considered statistically significant.

## 3. Results

The study cohort comprised 10 patients (M = 5, F = 5) with unilateral TMJ re-ankylosis with a mean age of 20.3 ± 1.37 years. The majority were Sawhney type IV (9/10) and affected the right joint (6/10), with a mean duration of ankylosis of 11.8 years. A narrow 40 mm size condyle component with a small fossa was used in 9/10 joints. Statistically significant improvement in MIO, increasing from a mean of 1.83 mm preoperatively to 37.83 mm (*p* < 0.001) at a mean follow-up of 60 months.

Preoperative bite force was 0 kg, which increased progressively to a statistically significant mean of 10.31 kg (*p* < 0.001) at 60 months, subsequently improving masticatory efficiency from a liquid diet preoperatively to a soft and regular diet in 3 to 6 months, progressing towards a hard diet at 12 months follow-up.

Protrusion and lateral excursion increased from a mean of 0 to 2.17 mm, 1.67 mm (right) and 1.33 mm (left), respectively. Notably, lateral excursion was more pronounced towards the prosthetic side when compared to the contralateral side. Ipsilateral jaw deviation towards the prosthetic side upon jaw opening was a consistent finding at 60 months follow-up.

**Figure 5:**
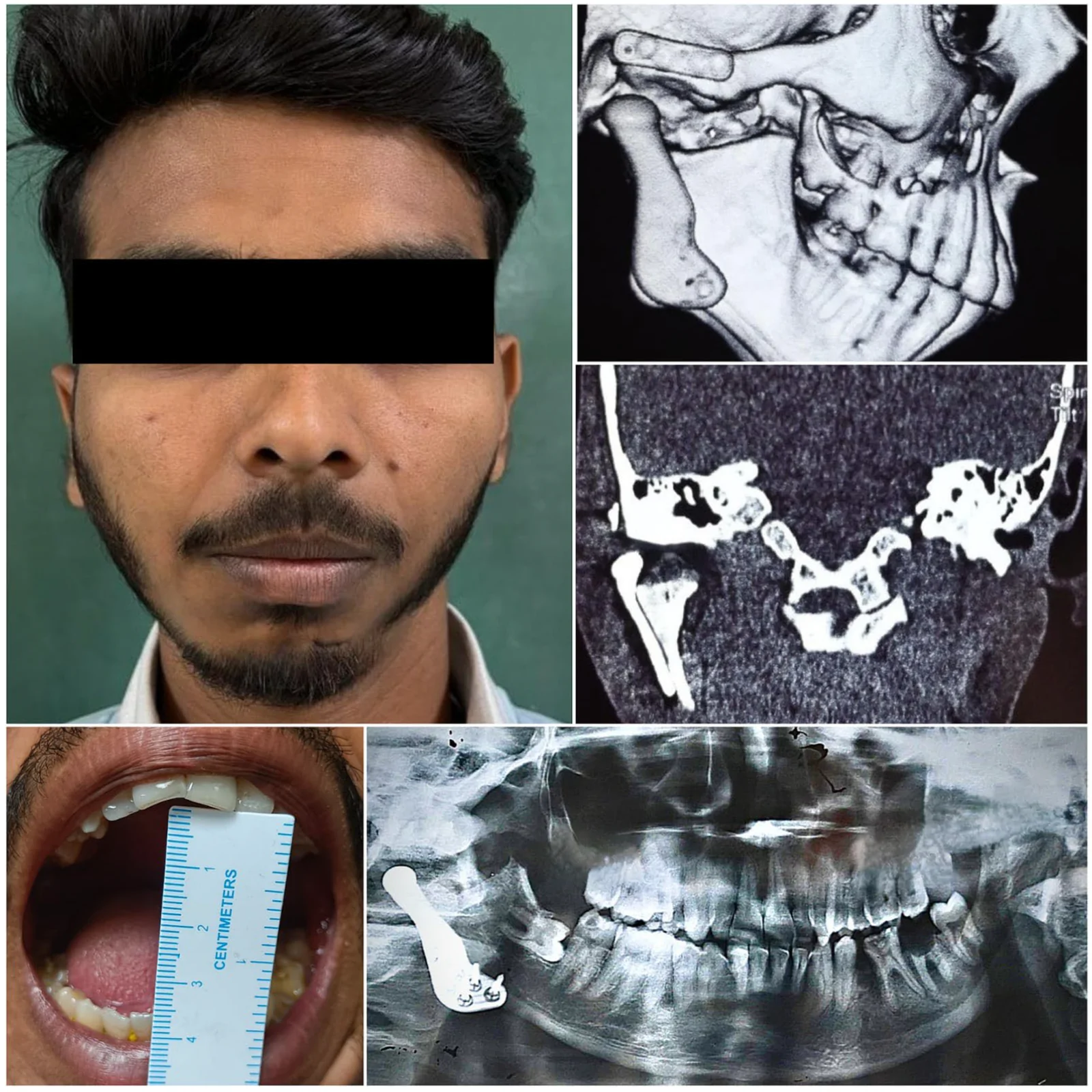
Sixty-month postoperative clinical and radiographic image showing acceptable functional outcome.

Facial nerve motor performance was tracked serially using the House–Brackmann (HB) grading scale. Preoperatively, 100% of subjects exhibited normal facial nerve function (HB Grade I). Immediately postoperatively, traction neuropraxia was observed, presenting as HB Grade II (mild dysfunction) in 33.3% and HB Grade III (moderate dysfunction) in 50.0%, while 16.7% retained Grade I function. Functional recovery occurred progressively: by 2 weeks, 83.3% had recovered to Grade II; by 4 weeks and 3 months, 83.3% had fully normalized to Grade I (with 16.7% in Grade II); and by 6 months and 1 year, 100% of patients achieved complete normalization (HB Grade I) with zero permanent motor deficits. Subjective self-reported questionnaire recorded statistically significant improvement in QoL [Figure 5]. Complications summarized in Table 2.

## 4. Discussion

TMJ re-ankylosis management for long-term success presents significant challenge due to disrupted anatomy, scar tissue and poor vascularity which undermines the efficacy of autogenous grafts, often leading to significant functional impairment, facial deformity, and reduced QoL. Marx and Mercuri found scar tissue thickness (≈440 μm) exceeds capillary penetration limits (180–220 μm), compromising graft viability [5], supporting the rationale for alloplastic TJR. TJR has emerged as a promising surgical option for managing such complex cases, particularly when conventional methods fail to restore joint function or prevent recurrence.

Incorporating VSP within a stock alloplastic reconstruction framework provides distinct clinical and safety advantages over conventional unguided stock placement. In TMJ re-ankylosis, distorted bony architecture, severe scarring, and loss of landmarks can challenge the reproducibility of VSP. Given that custom TJR allows virtually no margin for intraoperative adjustments, even minor surgical deviation from VSP can jeopardize prosthesis stability. This led to the thought of incorporating VSP with alloplastic stock TJR in the management of TMJ re-ankylosis. VSP enables precise delineation of the ankylotic mass, proximity to vital skull-base landmarks, and digital determination of resection planes. As highlighted in recent computational advancements, digital planning based on point-cloud filtering and geometric constraints significantly improves the spatial accuracy of TMJ prosthesis seating [6]. Furthermore, stock condylar plates possess fixed planar geometry that does not conform to the irregular, hyperostotic lateral ramal surface typical of re-ankylosis. Through digital 3D topographical analysis, areas of ramal cortical step-offs (>1.0 mm) were pre-emptively mapped for osteoplasty. This eliminated intraoperative guesswork, reduced operating room duration, and ensured 100% component sizing accuracy when translated to physical mock surgery and actual surgical execution, enhancing procedural efficiency and reducing intraoperative dubiety.

TMJA patients experience significant physical and psycho-social impact due to restricted MIO. In our study, statistically significant improvement in MIO is consistent with Leandro et al. [3], who reported 1-year postoperative MIO improvements from 11.3 to 28.1 mm in 300 patients. Bite force reflects masticatory system integrity, influenced by jaw muscle coordination and craniomandibular biomechanics. In TMJ re-ankylosis, long-standing joint immobility results in severe disuse atrophy of the masticatory apparatus. During TJR, functional reattachment of the lateral pterygoid and deep elevator fibers to the alloplastic prosthesis is not surgically successful, leading to altered kinematics [7]. Nevertheless, incisor bite force in our series improved significantly from 0 kg preoperatively to a mean of 10.31 kg (*p* < 0.001) in 12-month follow-up. This gain is attributable not to prosthetic muscle re-insertion, but rather to the restoration of a stable, rigid craniomandibular fulcrum (Class III lever system) and the alleviation of chronic mechanical limitation. The presence of a painless, stable prosthetic joint facilitates progressive neuromuscular rehabilitation, progressive recruitment, and compensatory hypertrophy of the intact contralateral masticatory sling and residual ipsilateral elevator muscles during functional chewing cycles. This progressive muscular adaptation supported the transition from a liquid diet to a solid, regular diet within 12 months.

Normal mandibular function comprises 38–50 mm mouth opening, 7–10 mm lateral excursion, 8–12 mm protrusion and 29–37.5° rotation [8]. In our study, modest improvements in lateral and protrusive movements were observed due to limited translational capacity, with motion occurring primarily via rotation. To address this, the prosthetic fossa is designed to lower the rotational axis, creating pseudo-translation [9]. Lateral excursion was greater toward the prosthetic side, likely due to detachment of the lateral pterygoid and pterygo-masseteric sling. This aligns with Linsen et al. [10] and Van Loon et al. [11], who observed similar post-TMJR mandibular kinematics and deviations. Analytical comparison with literature summarized in Table 3.

TMJ surgery, while offering therapeutic benefits, may also present potential complications. These include pain, hemorrhage, neurological alterations, hypersensitivity reactions, PJI, prosthesis dislocation, hypertrophic bone formation, re-ankylosis and metallosis. Facial nerve function was normal preoperatively. Postoperatively, all patients exhibited mild to moderate neuropraxic deficits in the temporal and marginal mandibular branches, with full recovery achieved by 1 year, mirroring SK Roy Chowdhary et al. [12] observations. According to Wolford et al. [13], hypersensitivity reactions and metallosis occur more frequently with metal-to-metal prostheses than with metal-on-UHMWPE. TMJ re-ankylosis remains a severe outcome, with heterotopic bone seen in ~50% of cases. Fat grafting, as suggested by Wolford [14] and Mercuri [15], shows preventive benefits. Roychoudhury et al. [16] documented a successful protocol using piezo-surgery, irrigation, fat grafting, and physiotherapy with no recurrence and PJI in 54 stock alloplastic TJR cases. Giannakopoulos et al. [17] reported a failure rate of 3.2% primarily attributed to infection or heterotopic bone formation, and Sahdev et al. [18] reported heterotopic bone formation in 3 out of 108 patients. Despite acknowledging the potential for complications, our study recorded no complications.

Patient-reported outcome measures questionnaire during follow-up provided subjective evaluation of overall improvement in MIO, mastication, diet, physical and psychosocial well-being, and functional rehabilitation post-surgery, in turn improving patients’ QoL.

Single-center prospective series with limited sample size and relatively short follow-up, no comparative group, with operative time logs being the limitations of this study. Future multi-center investigations with comparative cohorts will help further delineate the role of this workflow. The cost-effectiveness, rapid availability and intraoperative flexibility of stock prosthesis, while maintaining accuracy via mock-verified VSP, underscores the clinical efficacy of alloplastic stock TJR for TMJ re-ankylosis.

## 5. Conclusions

The dual-validation protocol—integrating virtual surgical planning with physical mock surgery on 3D models—provides a predictable, reproducible, and cost-effective clinical alternative to custom prostheses in the management of complex TMJ re-ankylosis. This protocol successfully combines the micro-anatomical accuracy of digital planning with the intraoperative flexibility and immediate availability of stock components. Over a 60-month follow-up, this workflow achieved substantial gains in patients’ QoL and functional rehabilitation, without long-term complications or disease recurrence. Further multicentric research involving larger cohorts and extended follow-up periods is recommended for standardization of this workflow.

## Figures and Tables

**Table 1:** Baseline data of subjects included in the study.

SL No.	Age (Years)/Sex *	TMJ Re-Ankylosis Side	Sawhney Type	No. of Previous Surgeries (#)	Mean Duration of Re-Ankylosis (Years)	MIO
1.	19/F	Right	Type IV	1 (GA)	12	1 mm
2.	18/M	Right	Type IV	1 (GA)	11	0 mm
3.	19/F	Right	Type IV	2 (IA)	10	0 mm
4.	19/M	Right	Type IV	2 (IA)	12	0 mm
5.	22/M	Left	Type III	1 (GA)	9	10 mm
6.	19/F	Left	Type IV	2 (GA)	13	0 mm
7.	28/F	Left	Type IV	2 (IA)	19	0 mm
8.	20/M	Right	Type IV	2 (IA)	11	1 mm
9.	21/F	Left	Type IV	1 (GA)	10	0 mm
10.	18/M	Right	Type IV	1 (GA)	11	1 mm

* M—Male, F—Female, # GA—Gap Arthroplasty, IA—Interpositional Arthroplasty.

**Table 2:** Comprehensive summary of complications across 60 months follow-up (*n* = 10).

Complication	Immediate Post-Op (*n*,%)	3 Months (*n*,%)	12 Months (*n*,%)	60 Months Follow-up (*n*,%)
Periprosthetic Joint Infection (PJI)	0 (0%)	0 (0%)	0 (0%)	0 (0%)
Hardware Loosening/Screw Fracture	0 (0%)	0 (0%)	0 (0%)	0 (0%)
Facial Nerve Neuropraxia (HB Grade II/III)	7 (70%)	1 (10%)	0 (0%)	0 (0%)
Heterotopic Bone Formation/Re-ankylosis	0 (0%)	0 (0%)	0 (0%)	0 (0%)
Prosthetic Dislocation/Subluxation	0 (0%)	0 (0%)	0 (0%)	0 (0%)
Allergic/Foreign Body Reaction	0 (0%)	0 (0%)	0 (0%)	0 (0%)

**Table 3:** Analytical comparison with existing literature.

Parameter	Present Study (Dual-Validation Stock)	Roychoudhury et al./Leandro et al. (Stock TJR)	Custom TJR Series (Mercuri, TMJ Concepts)
Cohort Type	Re-ankylosis (90% Type IV)	Ankylosis/Mixed Degenerative	End-stage osteoarthritis/Ankylosis
Mean MIO Gain	+36.0 mm (1.83 to 37.83)	+16.8 to +30.5 mm	+18.0 to +34.0 mm
Bite Force Gain	0 to 10.31 kg (*p* < 0.001)	Rarely reported quantitatively	Normalized over 24–36 months
Complications	0% PJI, 0% Re-ankylosis	PJI: 0–3.2%; Re-ankylosis: 2–5%	PJI: 1.5–3.0%

## Data Availability

The data presented in this study are available on request from the corresponding author due to the unavailability of any repository.
